# Recombinant follicular stimulating hormone plus recombinant luteinizing hormone versus human menopausal gonadotropins- does the source of LH bioactivity affect ovarian stimulation outcome?

**DOI:** 10.1186/s12958-021-00853-7

**Published:** 2021-12-09

**Authors:** M. Kirshenbaum, O. Gil, J. Haas, R. Nahum, E. Zilberberg, O. Lebovitz, R. Orvieto

**Affiliations:** 1 Department of Obstetrics and Gynecology, Chaim Sheba Medical Center (Tel Hashomer), Ramat Gan, Israel; 2 Sackler Faculty of Medicine, Tel Aviv University, Tel Aviv, Israel; 3 The Tarnesby-Tarnowski Chair for Family Planning and Fertility Regulation, at the Sackler Faculty of Medicine, Tel-Aviv University, Tel Aviv, Israel

**Keywords:** Assisted reproductive technology, Gonadotropins, Human chorionic gonadotropin, Luteinizing hormone, Ovarian stimulation

## Abstract

**Objective:**

Luteinizing hormone (LH) and human chorionic gonadotropin (hCG) activate distinct intracellular signaling cascades. However, due to their similar structure and common receptor, they are used interchangeably during ovarian stimulation (OS). This study aims to assess if the source of LH used during OS affects IVF outcome.

**Patients and methods:**

This was a cross sectional study of patients who underwent two consecutive IVF cycles, one included recombinant follicular stimulating hormone (FSH) plus recombinant LH [rFSH+rLH, (Pergoveris)] and the other included urinary hCG [highly purified hMG (HP-hMG), (Menopur)]. The OS protocol, except of the LH preparation, was identical in the two IVF cycles.

**Results:**

The rate of mature oocytes was not different between the treatment cycles (0.9 in the rFSH+rLH vs 0.8 in the HP-hMG, *p* = 0.07). Nonetheless, the mean number of mature oocytes retrieved in the rFSH+rLH treatment cycles was higher compared to the HP-hMG treatment cycles (10 ± 5.8 vs 8.3 ± 4.6, respectively, *P* = 0.01). Likewise, the mean number of fertilized oocytes was higher in the rFSH+rLH cycles compared with the HP-hMG cycles (8.5 ± 5.9 vs 6.4 ± 3.6, respectively, *p* = 0.05). There was no difference between the treatment cycles regarding the number of top-quality embryos, the ratio of top-quality embryos per number of oocytes retrieved or fertilized oocytes or the pregnancy rate.

**Conclusion:**

The differences in treatment outcome, derived by different LH preparations reflect the distinct physiological role of these molecules. Our findings may assist in tailoring a specific gonadotropin regimen when assembling an OS protocol.

## Condensation

Patients treated with rFSH+rLH yielded significantly higher numbers of mature oocytes and fertilized oocytes, with non-significantly lower pregnancy rate per transfer compared to those treated with HP-hMG.

## Introduction

Ovarian stimulation (OS) is a fundamental step in the process of artificial reproductive technology (ART). Administration of exogenous gonadotropins enables the recruitment and development of multiple follicles, in order to yield an optimal number of mature oocytes. The action of both Follicular stimulating hormone (FSH) and luteinizing hormone (LH) is required for follicular growth [1]. While FSH is the main regulator of follicular recruitment and genesis by stimulating granulosa cell proliferation and differentiation, the benefit of LH treatment may be derived from its effect on the synthesis of follicular steroids. Moreover, LH exerts an antiapoptotic effect on granulosa cells and promotes a paracrine signaling involved in cell expansion and oocyte maturation during folliculogenesis [2].

The first available source of LH was produced from the urine of postmenopausal women, known as human menopausal gonadotropin (hMG), which contained a mixture of FSH, human chorionic gonadotropin (hCG) and LH in varying amounts [3]. Improvement in purification techniques enabled the development of highly purified- hMG (HP-hMG) containing 1:1 ratio of FSH and LH bioactivity that is predominantly derived from hCG, since LH molecules are lost during the purification process. Another source of commercial gonadotropin is recombinant gonadotropin preparations. These include recombinant FSH (rFSH), rLH, rhCG and combined product of rFSH and rLH in a 2:1 ratio [4].

hCG and LH are structurally similar molecules, composed of two heterodimeric glycoprotein subunits, a mutual alfa subunit and a distinct beta subunit, which differs in length, post-translational glycosylation and structure [5]. Considering LH and hCG similar structure, their common receptor (LH/CGR) and data suggesting that hCG has a role throughout the menstrual cycle, they are currently used interchangeably in ART protocols to drive OS. Nonetheless, in vitro studies revealed specific bioactivity effects of LH and hCG, which differ by triggering separate intracellular signaling cascades. LH binding to the LH/CG receptor results in a more potent activation of the proliferative and anti-apoptotic pathways, whereas hCG has a higher potency for activation of the steroidogenic pathways [5–7].

Although the physiologic role of LH during the follicular phase of natural cycle is unequivocal, the importance of exogenous preparations with LH bioactivity during OS in normo-gonadotrophic women is of great debate. Previous randomized controlled trials and meta-analysis have demonstrated the benefit of adding different LH bioactivity preparations to FSH during OS, compared to FSH treatment only. Treatment with HP-hMG resulted in higher proportion of top- quality embryos developed, compared with rFSH. Moreover, at the end of stimulation, higher estradiol and lower progesterone levels were measured in the HP-hMG cycles compared with the rFSH cycles. A trend towards higher ongoing pregnancy rate and live birth rate was observed in the fresh HP-hMG cycles versus rFSH alone [8–10]. A systemic review assessing the role of rLH supplementation in OS in specific subgroups of patients demonstrated its benefit in women with normal ovarian reserve parameters and a hypo-response to OS and in women 36–39 years of age [11]. The superiority of HP-hMG or the combination of rLH with rFSH over rFSH alone during OS is thought to be attributed to the LH activity, which induces differences in the synthesis of follicular steroids, impacts oocyte maturation and improves embryo quality and endometrial receptivity [1, 12, 13].

In clinical practice, it is essential to consider all aspects when choosing the most appropriate gonadotropin therapy for OS. Since hCG and LH activate different physiological events, different commercially available LH preparation (hMG and rFSH+rLH) may affect the stimulation characteristics and consequently impact clinical outcomes. Nonetheless, rLH and hMG are still considered equivalent in clinical terms and there are no specific tools to guide the clinician in choosing the proper LH activity preparation for OS. A recent analysis which appraised prospective and retrospective studies comparing hMG and rFSH+rLH concluded that there is insufficient evidence to form any certain conclusions in favor of a particular source of preparation containing LH activity [14].

The current study was designed to compare OS outcome of two commercially available preparations with different source of LH bioactivity: rFSH+rLH in a fixed 2:1 ratio (Pergoveris®, Merck, Darmstadt, Germany) and HP-hMG, containing urinary FSH and LH activity provided by hCG in a fixed 1:1 ratio (Menopur®, Ferring pharmaceuticals).

## Patients and methods

We reviewed the computerized files of all consecutive women admitted to our IVF unit at the Sheba Medical Centre between March 2019 and March 2021. Inclusion criteria included patients who underwent two consecutive IVF cycles using the flexible multiple dose GnRH antagonist, where one cycle included r-FSH and r-LH in a 2:1 ratio (Pergoveris) and the other included urinary hCG, HP-hMG, (Menopur).

OS was started on cycle day 2–3, and was monitored by ultrasound and estradiol and progesterone levels from stimulation day 5 onwards. Once a leading follicle reached 13–14 mm, and/or the estradiol levels exceeded 400 pg/ml, co-treatment with GnRH antagonist was started. When the ovarian response was adequate, defined as at least three follicles measured above 17 mm, a trigger for ovulation was administered using hCG, GnRH agonist or both. Routine IVF or intra-cytoplasmic sperm injection (ICSI) were performed as accepted.

The initial gonadotropin dosage and the medication for ovulation trigger were determined by the treating physician and according to the patients’ clinical characteristics, ovarian reserve tests and the indication for fertility treatment. The elimination of bias in this selection, for the purposes of this study, was achieved by including only patients using the same initial FSH dosage and the same ovulation trigger and mode of fertilization during the two consecutive treatment cycles. The time period between the two cycles was up to 3 months.

Data regarding patients’ demographic and clinical characteristics and the response to OS and IVF treatment related variables were collected from the computerized clinical files. Primary outcome measure was the rate of mature oocytes retrieved. Secondary outcome measures were the duration of stimulation, total dosage of gonadotropin used, number of mature and fertilized oocytes, number of top-quality embryos, defined as embryos with ≥7 blastomeres and < 10% fragmentation on day-3 and pregnancy rate defined as positive beta-hCG test performed 2 weeks after an embryo transfer.

Statistical analysis was performed using the Statistical Package of Social Science version 27 (IBM Corp., USA). Categorical variables are presented as number of cases and percentage. Continuous data are presented as mean and standard deviation or median and inter-quartile range depending on normality test, as appropriate. Comparison of categorical variables were analyzed by Mcnemar’s test, and continuous variables by paired Students’ t-test, as appropriate. A two tailed *p*-value of < 0.05 was considered statistically significant.

Sample size calculation was based on a recent analysis comparing HMG and recombinant FSH plus LH during IVF cycles [14], using the rate of mature oocytes per cycle as the primary end point. Based on the expected difference of 7.2% between the rate of mature oocytes in the different treatment cycles, and a standard deviation of 19.9 in the rFSH+rLH treatment cycles and 16.2 in the HP-hMG treatment cycles, 53 patients were required to detect a significant difference with 80% power and 5% type 1 error.

The study was approved by the institutional review board of Sheba medical center (SMC-19-6610).

## Results

The study included 53 patients who underwent two consecutive IVF cycles, each with a different LH activity preparation, one included rLH and the other HP-hMG. Demographic and basic clinical characteristics are presented in Table 1. In 18 (35.8%) patients, the first cycle included rFSH+rLH treatment, which was converted to HP-hMG in the second, consecutive cycle.

**Table 1:** Basic characteristics of the study cohort and fertility treatment

	*n* = 53
Age	34.5 ± 5.5
BMI	22.7 ± 5.9
FSH	7.4 (6,9)
LH	4.7 (3,7)
FSH to LH	1.7 ± 0.8
Infertility treatment indication	
Fertility preservation	21 (39.6)
Poor ovarian reserve	3 (5.6)
PGT	14 (26.4)
Male factor	7 (13.2)
Tubal factor	2 (3.7)
Unexplained	4 (7.5)
Other	2 (3.7)
Final follicular maturation	
hCG	7 (13.2)
GnRH agonist	32 (60.3)
Dual trigger^a^	14 (26.4)
Pergoveris first cycle	18 (35.8)

Data are presented as mean ± SD, median (intra-quartile range) or numbers and percentage

^a^Dual trigger- hCG + GnRH agonist

The stimulation characteristics of the two cycles are presented in Table 2. There were no differences regarding the duration of stimulation or the total dose of FSH needed for stimulation between the two treatment cycles. As expected, the dose of LH in the treatment cycles including rFSH+rLH was significantly lower compared with the HP-hMG treatment cycles, due to the content of the medication which contains rFSH and rLH in a 2:1 ratio.

**Table 2:** Ovarian stimulation outcome

	MENOPUR	PERGOVERIS	*P* value
Days of stimulation	10.2 ± 1.6	10.4 ± 1.4	0.5
Total FSH	3210 ± 1484	3169 ± 1448	0.7
Total LH	2498 ± 1617	1243 ± 775	< 0.01
E2 level on trigger day (pmol/L)	7191 ± 4078	8129 ± 5330	0.1
P level on trigger day (nmol/L)	2.1 ± 1	2.5 ± 1.4	0.01
Endometrial thickness on trigger day (mm)	9.7 ± 2.8	9.7 ± 2.3	0.4
Number of follicles> 13 mm on trigger day	9.3 ± 4.4	11.5 ± 6.7	0.03
Number of oocytes	10.4 ± 5.5	11.5 ± 6.1	0.12
Number of M2 oocytes	8.3 ± 4.6	10 ± 5.8	0.01
Rate of M2 oocytes	0.8 ± 0.2	0.9 ± 0.13	0.07
Number of 2 PN	6.4 ± 3.6	8.5 ± 5.9	0.05
Number of top-quality embryos (day 3)	3.4 ± 3	4.0 ± 3	0.2
Rate of top-quality embryos (TOP/oocyte)	0.3 ± 0.2	0.3 ± 0.2	0.9
Rate of top-quality embryos (TOP/2PN)	0.5 ± 0.3	0.5 ± 0.3	0.7

Data are presented as mean ± SD

*PN* Pronuclei

Estradiol levels at the day of ovulation trigger were comparable between the two groups. Nonetheless, the progesterone levels at the trigger day were significantly higher in the rFSH+rLH cycles compared with the HP-hMG cycles (2.5 ± 1.4 nmol/L vs 2.1 ± 1 nmol/L respectively, *p* < 0.01).

The rate of mature oocyte did not differ between the groups (0.9 in the rFSH+rLH cycles vs 0.8 in the HP-hMG cycles, *p* = 0.07). Nonetheless, the mean number of mature oocytes retrieved in the rFSH+rLH treatment cycles was significantly higher compared to the HP-hMG treatment cycles (10 ± 5.8 vs 8.3 ± 4.6, respectively, *P* = 0.01). Likewise, the mean number of fertilized oocytes was significantly higher in the rFSH+rLH cycles compared with the HP-hMG cycles (8.5 ± 5.9 vs 6.4 ± 3.6, respectively, *p* = 0.05).

There was no difference between the treatment cycles regarding the number of top-quality embryos nor the ratio of top-quality embryos to the number of oocytes retrieved or fertilized oocytes (Table 2). The pregnancy rate per fresh transfer was higher among the HP-hMG treatment cycles (4/14, 29%) compared with the rFSH+rLH cycles (2/14, 15%), although this difference did not reach statistical significance (*p* = 0.3).

## Discussion

In the present study, we compared the OS outcomes of two LH activity preparations- recombinant LH and HP-hMG, in which the LH activity is derived mostly from hCG. Patients treated with rFSH+rLH yielded significantly higher numbers of mature oocytes and fertilized oocytes, with non-significantly lower pregnancy rate per transfer (15% vs 29%, respectively. *P* = 0.3), compared to those treated with HP-hMG.

LH and hCG are heterodimeric glycoprotein hormones sharing approximately 85% structural identity. Both of them bind to a mutual LH/choriogonadotropin receptor (LH/CGR), a G protein-coupled receptor with an extra-cellular binding domain to which LH and hCG bind in distinct, specific regions. The distinct molecular structure of the beta-LH and beta-hCG subunits results in different conformational changes of the LH/CGR, leading to activation of different intracellular cascades [5]. The post receptor, intra-cellular reactions, include an activation of adenylate cyclase, which increases the intracellular pool of cAMP and results in steroidogenesis, and an extracellular signal-regulated protein kinases 1 and 2 (ERK1/2) and AKT, with putative roles in cell proliferation, differentiation and survival [7]. In vitro studies found that hCG is five times more potent than LH with respect of cAMP production and steroidogenesis pathway, while LH strongly activates ERK1/2 and AKT pathways, thus induces proliferation and anti-apoptotic effect [6, 15]. Although in vitro models provided the evidence of hormone- specific actions, whether they influence differently on in vivo OS response remains unclear and published evidence of the difference between rLH and hCG during OS is surprisingly scarce.

The main findings of our study were higher number of mature oocytes retrieved and higher number of oocytes fertilized in the rFSH+rLH compared with the HP-hMG treatment cycles. These differences confirm that rLH and hCG poses different influences also in vivo during OS cycles. The higher number of mature oocytes may be due to the synergistic effect of FSH and LH on follicular growth, proliferation and maturation. Moreover, the addition of LH shifts granulosa cells from proapoptotic to proliferative pathways, in contrast to hCG which has proapoptotic effect mediated by relatively higher intracellular cAMP concentrations [16, 17]. Gomez-Palomares et al. evaluated the OS outcomes in women over 38 years old supplemented with rFSH+rLH or hMG [18]. Similar to our results, they found a higher rate of mature oocytes in the group treated with rFSH+rLH. Pacchiarotti et al. in their randomized controlled trial comparing rFSH+rLH with hMG, also found higher number of oocytes retrieved and higher number of mature oocytes in the rFSH+rLH cycles, although the rate of matured oocytes per overall oocytes yield was higher in the hMG cycles [19]. Unlike our study, that included down regulation by GnRH antagonist, Pacchiarotti et al. used the long GnRH agonist down regulation protocol, which may influence differently the initial follicular reaction to rLH or hCG.

When comparing the IVF treatment outcomes of rFSH+rLH to HP-hMG, we must also consider the differences in the type of FSH molecules, which may be related to the variation observed between the two treatments. The higher mature oocytes yield demonstrated in the rFSH+rLH treatment cycles, may be derived from the greater effectiveness of the rFSH isoform compared with urinary FSH, rather than from the effect induced by rLH, as it is well-established that rFSH leads to higher follicular recruitment compared with HP-hMG [1, 8, 20].

Another interesting finding of our study was higher progesterone levels in the rFSH+rLH cycles compared with the HP-hMG cycles. This finding might be explained by the higher number of follicles in the rFSH+rLH treatment cycles. However, we must also consider the different effect of rLH and hCG on the steroidogenesis process, as an alternative explanation for the different endocrine profile. Previous studies comparing progesterone levels following OS with rFSH or HP-hMG have revealed similar findings; rFSH stimulation alone was associated with higher progesterone levels at the end of stimulation, even after adjusting for ovarian response [1, 8, 21, 22]. In accordance to our study, a study by Sebag-Peyrelecade et al. which compared the progesterone levels of rFSH+rLH treatment with those of hMG treatment, revealed that supplementation of rLH was not sufficient to decrease the progesterone levels to those observed in the hMG treatment cycles, unrelated to the degree of ovarian response [21]. Therefore, we may deduce that the lower progesterone levels measured in the hMG cycles are attributed to the hCG content.

In the present study, we did not find any differences regarding the rate of mature oocytes nor the number of top-quality embryos between the rFSH+rLH and HP-hMG treatments, despite of the initial higher number of fertilized oocytes in the rFSH+LH treatment cycles. This observation suggests that although rFSH+rLH is more potent in follicular genesis and oocytes yield, HP-hMG may favor embryonal maturation. In accordance with our findings, previous studies that compared the use ofHP-hMG vs rFSH, such as the MERiT and the MEGASET trials, have found that HP-hMG results in higher proportion of top-quality embryos [8, 20].

Of note, although not statistically significant, the pregnancy rate per fresh transfer was higher among the HP-hMG treatment cycles compared to the rFSH+rLH cycles. This difference in pregnancy rate may be a consequence of the higher progesterone levels during the rFSH+rLH cycles, which negatively influence the endometrial receptivity and the essential synchronization between the embryo and the endometrium. Nonetheless, because the present study is not sufficiently powered to detect differences in pregnancy rate, this data should be viewed with caution.

A major strength of our study is that we compared the different LH activity preparations in the same cohort of patients. The fact that all women that participated in our study had two consecutive treatment cycles using rFSH+rLH in one cycle and hMG in the other, helps to eliminate multiple bias factors and to attribute the study results to the different treatment preparations.

A limitation of our study is the lack of data regarding cumulative clinical pregnancy or live birth rates. Nonetheless, one may say that when the question of investigation is the effect of LH activity products on OS, the primary outcome has to be the first measurable parameter of gonadotropin influence, i.e., the ovarian response. Moreover, the majority of our study cohort comprised of patients who underwent IVF treatment for social fertility preservation. In this section of patients, we are most interested in achieving an optimal number of mature oocytes.

In conclusion, our study suggests that gonadotropins preparations have different influence on OS outcome, proving the necessity of tailoring a specific gonadotropin regimen when assembling a treatment protocol. rFSH+rLH resulted in higher number of matured oocytes and fertilized oocytes, while the lack of difference regarding the number of top-quality embryos between the preparations might suggest an encouraging effect of hMG on oocytes and embryo quality. These treatment characteristics, derived by different LH preparation, reflect the physiological role of these molecules as previously indicated by in vitro data. Further large follow up studies addressing the question whether rLH should be uniformly used in all IVF cycles or only selectively are required.

## Data Availability

All authors have a secured access to the study data. Data are available from the corresponding author on request.

## Abbreviations

ART: Artificial reproductive technology
FSH: Follicular stimulating hormone
hCG: Human chorionic gonadotropin
hMG: human menopausal gonadotropin
HP-hMG: highly purified- hMG
ICSI: Intra-cytoplasmic sperm injection
IVF: In-vitro fertilization
LH: Luteinizing hormone
OPU: Ovum pick-up
OS: Ovarian stimulation
rFSH: Recombinant FSH
